# Supplementary material for: Grazing Intensities Regulated the Effects of Seasonal Dietary Pattern on Gut Bacterial Community Composition of Sheep
Source: Microorganisms. 2025 Jun 14;13(6):1392. doi: 10.3390/microorganisms13061392 (PMC12196464; doi:10.3390/microorganisms13061392)
Supplement: Supplementary file 1 [file microorganisms-13-01392-s001.zip › microorganisms-3662621-supplementary.pdf]

Supplementary material

Table S1. Mean (± SE; *n* =3) above-ground biomass of plant families and select forbs across three months of the growing season for three grazing intensities (LG = light grazing; MG = moderate grazing; HG = heavy grazing).

Month	Plant Families	Above-ground Biomass (g·m^{-2})		
		LG	MG	HG
Jul.	Poaceae	108.54 ± 38.76	91.19 ± 6.65	85.73 ± 16.74
	Asteraceae	1.94 ± 1.42	0.71 ± 0.34	0.63 ± 0.38
	Cyperaceae	4.86 ± 1.97	4.46 ± 0.90	2.88 ± 0.74
	Liliaceae	0.89 ± 0.69	1.36 ± 1.27	0.35 ± 0.16
	Rosaceae	0.15 ± 0.13	0.12 ± 0.03	0.13 ± 0.18
	Fabaceae	2.79 ± 0.36	0.46 ± 0.04	0.53 ± 0.06
	Forbs	5.83 ± 2.77	0.36 ± 0.32	0.52 ± 0.35
Aug.	Poaceae	175.45^{a} ± 37.67	113.48^{ab} ± 21.85	82.35^{b} ± 16.31
	Asteraceae	5.73 ± 3.42	4.29 ± 2.94	5.32 ± 1.87
	Cyperaceae	7.61 ± 3.67	6.57 ± 2.16	4.13 ± 2.66
	Liliaceae	1.93 ± 1.68	2.06 ± 1.76	0.61± 0.48
	Rosaceae	1.61 ± 0.94	0.66 ± 0.07	1.12 ± 0.16
	Fabaceae	3.97 ± 3.80	0.40 ± 0.04	0.89± 0.33
	Forbs	6.78 ± 4.89	3.84 ± 0.66	2.75 ± 1.33
Sep.	Poaceae	127.96^{a} ± 13.02	102.09^{a} ± 11.51	80.24^{b} ± 13.93
	Asteraceae	3.86 ±2.48	1.85 ±1.11	1.90 ±0.70
	Cyperaceae	6.21 ±0.47	2.96 ±0.63	4.79 ±1.44
	Liliaceae	1.06^{a} ±0.07	0.75^{a} ±0.23	0.17^{b}±0.01
	Rosaceae	1.17 ±1.25	0.39 ±0.04	0.23 ±0.02
	Fabaceae	3.15^{a} ±2.18	0.43^{b} ±0.54	1.32^{ab} ±1.66
	Forbs	3.24 ±2.46	0.77 ±0.54	1.08 ±0.82

Means within a family and seasonal month followed by the same letter are not significantly different ($P > 0.05$) based on Tukey's post hoc test.

Table. S2. Mean (± Pooled SE; *n* =3) forage quality of plant families and select forbs across three months of the growing season for three grazing intensities (LG = light grazing; MG = moderate grazing; HG = heavy grazing).

Plant nutrient	Vegetation group	Month	LG	MG	HG	Pooled SE
Crude protein	Poaceae	Jul.	13.57^{A}	12.88	14.14	0.90
		Aug.	11.99 ^{A}	13.49	14.32	0.79
		Sep.	10.51 ^{B}	11.53	11.23	0.62
	Asteraceae	Jul.	13.80 ^{A}	13.89	14.52	0.32
		Aug.	13.7 ^{A}	14.10	16.15	0.50
		Sep.	11.99 ^{Bb}	11.73^{b}	14.25^{a}	0.53
	Cyperaceae	Jul.	13.32 ^{A}	13.69	12.17	0.53
		Aug.	12.47 ^{A}	14.79	13.63	0.47
		Sep.	10.73 ^{B}	10.88	11.44	0.41
	Liliaceae	Jul.	15.00	16.59	15.77	0.59
		Aug.	14.50	14.48	16.53	0.4
		Sep.	14.11	12.28	13.70	0.47
	Rosaceae	Jul.	14.38	15.01	14.43	0.20
		Aug.	15.78	15.38	14.00	0.35
		Sep.	14.36	14.74	13.85	0.34
	Fabaceae	Jul.	17.61 ^{A}	19.04	18.32	0.63
		Aug.	15.95 ^{AB}	18.33	17.96	0.66
		Sep.	12.74 ^{B}	12.51	10.44	0.51
	Forbs	Jul.	16.71 ^{A}	16.75	17.26	0.30
		Aug.	15.02 ^{B}	13.72	12.81	0.35
		Sep.	12.84 ^{C}	13.27	12.50	0.16
Neutral detergent fiber	Poaceae	Jul.	65.84	63.09	61.31	1.68
		Aug.	57.86	59.64	62.13	3.86
		Sep.	65.54	65.97	66.35	1.79
	Asteraceae	Jul.	51.87	50.05	49.40	2.15
		Aug.	48.65	48.95	42.11	1.80
		Sep.	45.11	46.90	49.39	2.37
	Cyperaceae	Jul.	66.86	62.15	58.88	2.32
		Aug.	64.72	45.82	62.95	4.00
		Sep.	63.35	62.16	59.21	1.50
	Liliaceae	Jul.	56.00 ^{A}	56.25	41.69	3.06

		Aug.	40.90 [B]	44.87	45.59	2.57
		Sep.	39.99 [B]	42.21	43.05	1.01
	Rosaceae	Jul.	32.50	36.74	37.40	1.89
		Aug.	34.99	37.22	35.76	1.76
		Sep.	40.39	43.06	33.59	1.43
	Fabaceae	Jul.	35.91[B]	36.41	46.60	2.33
		Aug.	39.32[B]	43.54	43.44	2.00
		Sep.	54.08 [Ab]	42.18[b]	65.03[a]	3.92
	Forbs	Jul.	43.31	48.79	43.08	2.02
		Aug.	38.98	45.81	37.50	3.17
		Sep.	46.19	39.40	43.46	2.32
Acid detergent fiber	Poaceae	Jul.	30.12	32.93	30.66	1.01
		Aug.	33.12	31.44	29.15	0.72
		Sep.	31.71	30.74	31.61	0.81
	Asteraceae	Jul.	36.42[A]	38.70	36.88	1.03
		Aug.	32.45[B]	33.34	31.29	0.85
		Sep.	31.61[B]	32.28	33.63	1.02
	Cyperaceae	Jul.	30.90	31.40	29.47	0.56
		Aug.	29.21	27.16	28.54	0.69
		Sep.	27.87	26.65	26.89	0.48
	Liliaceae	Jul.	34.98[A]	33.32	30.93	0.86
		Aug.	27.42[B]	32.75	27.72	1.77
		Sep.	27.71[B]	29.61	30.10	0.50
	Rosaceae	Jul.	21.86	21.52	14.11	1.29
		Aug.	22.28	23.79	21.48	0.88
		Sep.	24.58	21.84	18.66	1.00
	Fabaceae	Jul.	21.45	28.20	35.19	2.16
		Aug.	25.71	24.93	25.79	1.33
		Sep.	24.78	24.12	30.47	1.38
	Forbs	Jul.	26.22	36.23	34.80	1.72
		Aug.	30.56	27.12	26.82	1.11
		Sep.	32.72	23.76	27.80	1.99

Means within a forage quality variable and seasonal month with different lowercase letters indicate significant differences among grazing intensities ($P < 0.05$) and means within a forage quality variable and grazing intensity followed by different uppercase letters indicate significant differences among seasonal months ($P < 0.05$) based on Tukey's post hoc test.
